# Notch ligand Jagged1 promotes mesenchymal stromal cell-based cartilage repair

**DOI:** 10.1038/s12276-018-0151-9

**Published:** 2018-09-21

**Authors:** Junkui Sun, Zhengliang Luo, Guangxi Wang, Yuping Wang, Yisheng Wang, Margaret Olmedo, Massimo Max Morandi, Shane Barton, Christopher G. Kevil, Bing Shu, Xifu Shang, Yufeng Dong

**Affiliations:** 1 Department of Orthopaedics, the First Affiliated Hospital, Zhengzhou University, Henan, 450001 China; 2 Department of Orthopaedics, Louisiana State University Health Sciences Center, Shreveport, LA USA; 3 Department of Orthopedic Surgery, Anhui Provincial Hospital, 17 Lujiang Rd, Hefei, China; 4 Department of Obstetrics and Gynecology, Louisiana State University Health Sciences Center, Shreveport, LA USA; 5 Department of Pathology, Louisiana State University Health Sciences Center, Shreveport, LA USA; 6 Longhua Hospital, Shanghai University of Traditional Chinese Medicine, Shanghai, China

## Abstract

Placenta-derived mesenchymal stromal cells (PMSCs) provide a promising cell source for tissue regeneration. However, rapid induction of PMSC chondrogenic differentiation during therapeutic transplantation remains extremely challenging. Here we undertook a study to determine if Notch inhibition by soluble Jagged1 (JAG1) peptides could be utilized to accelerate PMSC-induced cartilage regeneration in a mouse post-traumatic osteoarthritis (PTOA) model. Our results showed that treatment of PMSCs with soluble JAG1 significantly enhanced chondrogenesis in culture as shown by increased alcian blue staining and decreased Notch target Hes1 expression when compared to those in lgG-treated control cells. Importantly, significantly enhanced cartilage formation and decreased joint inflammation were observed when JAG1-treated PMSCs were injected into mouse PTOA knee joints. Finally, in vivo cell tracing showed that more JAG1-treated PMSCs remained in knee joint tissues and that JAG1-treated PMSCs exhibited greater PMSC chondrogenic differentiation than lgG-treated control PMSCs at 4 weeks after injection. These data indicate that transient Notch inhibition by soluble JAG1 could be used to enhance PMSC survival and chondrogenic differentiation, thereby increasing the therapeutic potential of PMSCs for cartilage regeneration.

## Joint repair: Growing new cartilage from placental stem cells

Stem cells derived from placental tissue may help in treating damaged joints, thanks to an improved method for encouraging them to form cartilage. Placenta-derived stem cells are readily available, as the placenta is usually discarded after birth. These stem cells can easily be grown into cartilage tissue in a laboratory, but not when transplanted. Researchers led by Yufeng Dong (Louisiana State University Health Sciences Center, Shreveport, USA) and Xifu Shang (Anhui Provincial Hospital, Hefei, China) treated stem cells with a naturally occurring protein, JAG1, which blocks the signal that prevents them from forming cartilage. They found that JAG1 increased the stem cells’ capacity for cartilage formation. When JAG1-stimulated stem cells were injected into a mouse model of joint disease, cartilage formation was improved, and joint inflammation was reduced.

## Introduction

Mesenchymal stromal cells (PMSCs) derived from placental tissue are highly proliferative with multipotent differentiation capacity^1^. Compared to the widely studied embryonic or bone marrow-derived MSCs, PMSCs have several advantages, including no ethical issues as the placenta is a medical waste generally discarded after birth, large availability, and no invasive injury for the donor^2^. Therefore, PMSCs are an excellent cell source for regenerative medicine. Although most of the mesenchymal stem cells isolated from different sources are very similar in an undifferentiated state, differences in capacity for multilineage cell differentiation have also been observed. Previous data showed that PMSCs have a lower potential to undergo adipogenesis, while having a higher potential to undergo chondro-osteogenesis than other MSCs^3^. These findings provide a platform for further investigation of the in vivo efficacy of PMSCs for cartilage regeneration.

While PMSCs exhibit a capacity for chondrogenesis in vitro, without the stimulation of chondroinductive molecules, they are unable to spontaneously undergo chondrogenesis in vivo. To induce rapid PMSC chondrogenic differentiation, the effect of signaling pathways on stromal cell differentiation must be understood, especially the integrated signal inputs that initiate stromal cell chondrogenic differentiation^4^. Previous studies from our group demonstrated that Notch signaling is a significant factor in MSC maintenance, proliferation and lineage commitment^5^. Particularly, inhibition of Notch signaling significantly enhances limb bud-derived MSC chondrogenesis^6^. There are four Notch receptors (Notch1–Notch4) and six Notch ligands (Delta-like 1, 2, 4, Jagged 1, 2) that have been identified in mice and humans. Binding of these ligands to receptors leads to proteolytic cleavage and release of the Notch intracellular domain (NICD) from the plasma membrane, which translocates to the nucleus and regulates transcription of downstream targets, most notably the Hey and Hes genes^5,7^. Previous studies have demonstrated that the Notch ligand Jagged1 (JAG1) is abundantly expressed and markedly increased in cartilage from patients with osteoarthritis (OA), a disease characterized by destruction of articular cartilage due to progressive articular chondrocyte apoptosis^8^. Additionally, activation of Notch signaling in chondrocytes induces cell death in OA mice, while inhibition of Notch signaling in chondrocytes suppresses OA development in a murine surgical model^9^, suggesting that Notch signaling plays a crucial role in OA pathophysiology and articular cartilage homeostasis.

Although increased Notch activity is involved in OA development, its role as a therapeutic target to prevent or rescue injury-induced cartilage destruction still needs clarification. Since the soluble JAG1 ligand has previously been shown to inhibit endogenous Notch signaling in vitro^10,11^, here, we utilized the negative effect of the soluble form of JAG1 peptides on Notch signaling to further enhance the PMSC therapeutic effect on cartilage repair.

## Materials and methods

### Human PMSC isolation and culture

Placentas delivered by normal pregnant women were collected immediately after delivery. Since the placenta is considered a medical waste, no consent from the patients was needed. Collection of human placentas for MSC isolation was approved by the IRB at Louisiana State University Health Science Center – Shreveport (LSUHSC-S), and MSC isolation was processed at the Department of Gynecology and Obstetrics, LSUHSC-S. The procedures for PMSC isolation and culture were performed as described before^3^. Briefly, villous tissue was separated by sterile dissection from different cotyledons, excluding chorionic and basal plates. After extensive washing with ice-cold phosphate-buffered saline (PBS), villous tissue was digested with trypsin (0.125% trypsin solution containing 0.1 mg/ml DNase I and 5 mM MgCl2) in Dulbecco’s Modified Eagle’s Medium (DMEM) at 37 °C for 90 min. Digested cells were collected and cultured in DMEM supplemented with 10% fetal bovine serum (FBS). PMSCs started to grow in 3–5 days. At ~80% confluence, the cells were passaged with TrypLE™ Express (Invitrogen, Carlsbad, CA, USA). Passage 4 (P4) PMSCs were characterized by flow cytometry using the following antibodies: CD29-APC, CD73-PE, CD90-APC, and CD166-APC (Abcam, Cambridge, MA, USA). CD34-APC served as a negative control. The nontransmembrane, soluble forms of the JAG1 peptides encoding the Delta/Serrate/lag-2 domain (DSL) (CDDYYYGFGCNKFCRPR) (AnaSpec Inc., USA) were used to inhibit Notch signaling in PMSCs, and lgG peptides were used as the control.

### Luciferase assay

JAG1 peptide-coated plates were prepared as reported previously^12^, and PMSCs cultured in regular or JAG1-coated plates were then transfected with Notch-responsive RBPJ-Luc and SV40-Renilla-Luc in the presence of Lipofectamine 2000 (Invitrogen). Forty-eight hours after transfection and treatment, lysates were analyzed with a Dual Luciferase Assay Kit (Promega).

### Osteogenic and adipogenic differentiation

To induce PMSC osteogenic differentiation, P4 PMSCs were cultured with DMEM supplemented with 10% FBS, ascorbic acid (50 mg/ml), 1 μmol/L dexamethasone, and β-glycerophosphate (10 mM) for up to 14 days. Alkaline phosphatase (ALP) and alizarin red staining were performed to visualize osteogenic differentiation as previously described^13^. RNA was also extracted for osteogenic gene expression of Runx2 and Osteocalcin. Adipogenic differentiation was induced by medium containing insulin (10 mg/ml), dexamethasone (1 mM), and 3-isobutyl-1-methylxanthine (0.5 mM) for up to 21 days. Oil Red O staining, PPARγ and C/EBPa gene expression were used to monitor adipogenic differentiation.

### Chondrogenic cell pellet culture

PMSC pellets were obtained by centrifuging 2.5 × 10^4^ cells at 250 × g for 5 min in 15 ml polypropylene conical tubes as reported^12^. Pellets were cultured for 14 days in 0.5 ml of serum-free MesenCult™-Chondrogenic differentiation medium (Stem cell Technologies, Vancouver, Canada) containing soluble JAG1 (10 µg/ml) or lgG (10 µg/ml) peptides. Harvested pellets were fixed in 4% paraformaldehyde (PFA), dehydrated, and embedded in paraffin. Serial sections of 5 µm were stained with alcian blue/orange G. Immunohistochemical (IHC) analysis for Col-II was performed as described previously^12^.

### In vivo experiment

All mouse experiments were performed according to the protocol approved by the Animal Care and Use Committee of the Louisiana State University Health Sciences Center. The mouse experimental OA model was induced in the right knee joint by meniscal/ligamentous injury (MLI) based on previous studies^14,15^. Briefly, 36 C57BL6/J mice were anesthetized and shaved on right knee joints for aseptic surgery. The medial joint capsule adjunct to the patellar tendon was incised with a blade to expose the medial meniscotibial ligament and the entire medial meniscus. After carefully cutting off the medial meniscus and washing with saline to remove tissue debris, the medial capsular incision was then closed with sutures. For 4 weeks after MLI surgery, 8 μl PBS solution containing 0.2 million PMSCs mixed with JAG1 (10 µg/ml) or lgG (10 µg/ml) were injected (once a week for 4 weeks) into the joint cavity from the medial edge of the patellar ligament in each group (*n* = 12). To track the in vivo contribution of cells, PMSCs were labeled by red fluorescent dye using the Qtracker® 585 Cell Labeling Kit (Thermo Fisher Scientific). Mice were euthanized 4 weeks after intra-articular injection (8 weeks after MLI surgery).

Knee joint samples for histology were fixed in 4% formaldehyde, decalcified in 10% ethylene diamine-tetra acetic acid (EDTA) solution, and embedded in paraffin. Tissue sections were observed using a fluorescence microscope for labeled PMSCs and stained with H&E and alcian blue/orange G for histological scoring. In a blinded fashion, three examiners used a modified Osteoarthritis Research Society International (OARSI) score to evaluate the histological condition of the articular cartilage surface as described before^16^. In brief, each section was assigned a grade 0–6: 0, normal cartilage; 0.5, loss of alcian blue staining without structural changes; 1, small fibrillations without loss of cartilage; 2, vertical clefts down to the layer below the superficial layer; 3–6, vertical clefts or erosion to the calcified cartilage [<25% (grade 3), 25–50% (grade 4), 50–75% (grade 5) and >75% (grade 6) of the articular surface is affected]. The maximal score was used to represent severity of the OA progression for each mouse. Immunohistochemical (IHC) analysis for Col-II and Col-X was performed as previously described^12^. IHC for tracing human cells in knee joint tissue was also performed using anti-human nuclear antigen antibody (ab191181, Abcam, USA). Chondrocyte apoptosis in tibia articular cartilage was further determined by the Terminal deoxynucleotidyl transferase (TdT) dUTP Nick-End Labeling (TUNEL) assay using an in situ Cell Death Detection Kit (Roche Diagnostics, Mannheim, Germany). For synovial tissue RNA extraction, synovial membrane surrounding the knee joint was collected and homogenized in cold Trizol reagent (Life Technologies). Chloroform was mixed with the lysate and, following centrifugation, the aqueous RNA layer was transferred to a new microcentrifuge tube. Prechilled isopropanol was then mixed with the RNA layer. Following centrifugation, the RNA pellet was washed with 70% ethanol and redissolved in RNase-free water. The concentration/purity of the RNA sample(s) was measured using the NanoDrop 2000.

### Quantitative RT-PCR

Total RNA isolated from PMSCs, and synovial tissues was reverse transcribed to cDNA using an iScript cDNA Synthesis Kit (Bio-Rad, Hercules, CA, USA) according to the manufacturer’s instructions. β-actin was used as an internal control. For gene expressions, RT-PCRs were performed on ABI 7900HT fast Real-Time PCR System using primers for Notch target Hes1, chondrogenic (Col-II, Aggrecan and SOX9), osteogenic (Runx2 and Osteocalcin), adipogenic (PPARr and C/EBPa), and proinflammation (TNF-α, IL-1β, MMP-1, MMP-13) marker genes. All primer sequences are available upon request.

### Statistical analysis

The above experiments were repeated at least three times independently. All data are presented as the mean ± SD. Statistical significance among the groups was assessed using one-way analysis of variance (ANOVA). The level of significance was *P* < 0.05.

## Result

### Characterization of PMSCs

**Fig. 1:**
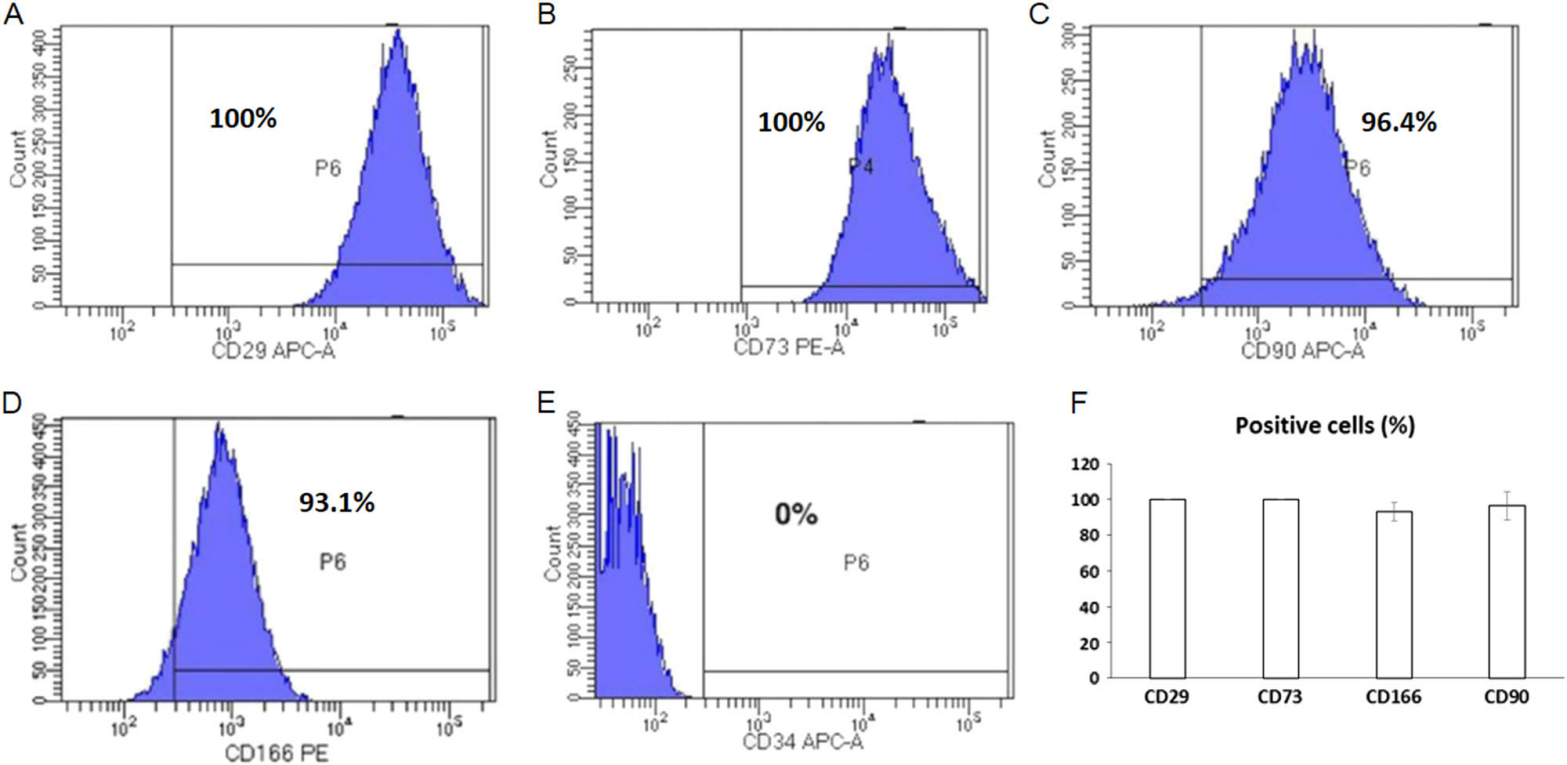
Characterization of human PMSCs. **a**–**e** Representative flow cytometry histograms showing CD29, CD73, CD90 and CD166-positive PMSCs and CD34-negative PMSCs in passage 4 cultures, respectively. **f** Quantified percentage of CD29, CD73, CD90 and CD166-positive MSCs in passage 4 cultures. Data are the means ± SD of three independent experiments

**Fig. 2:**
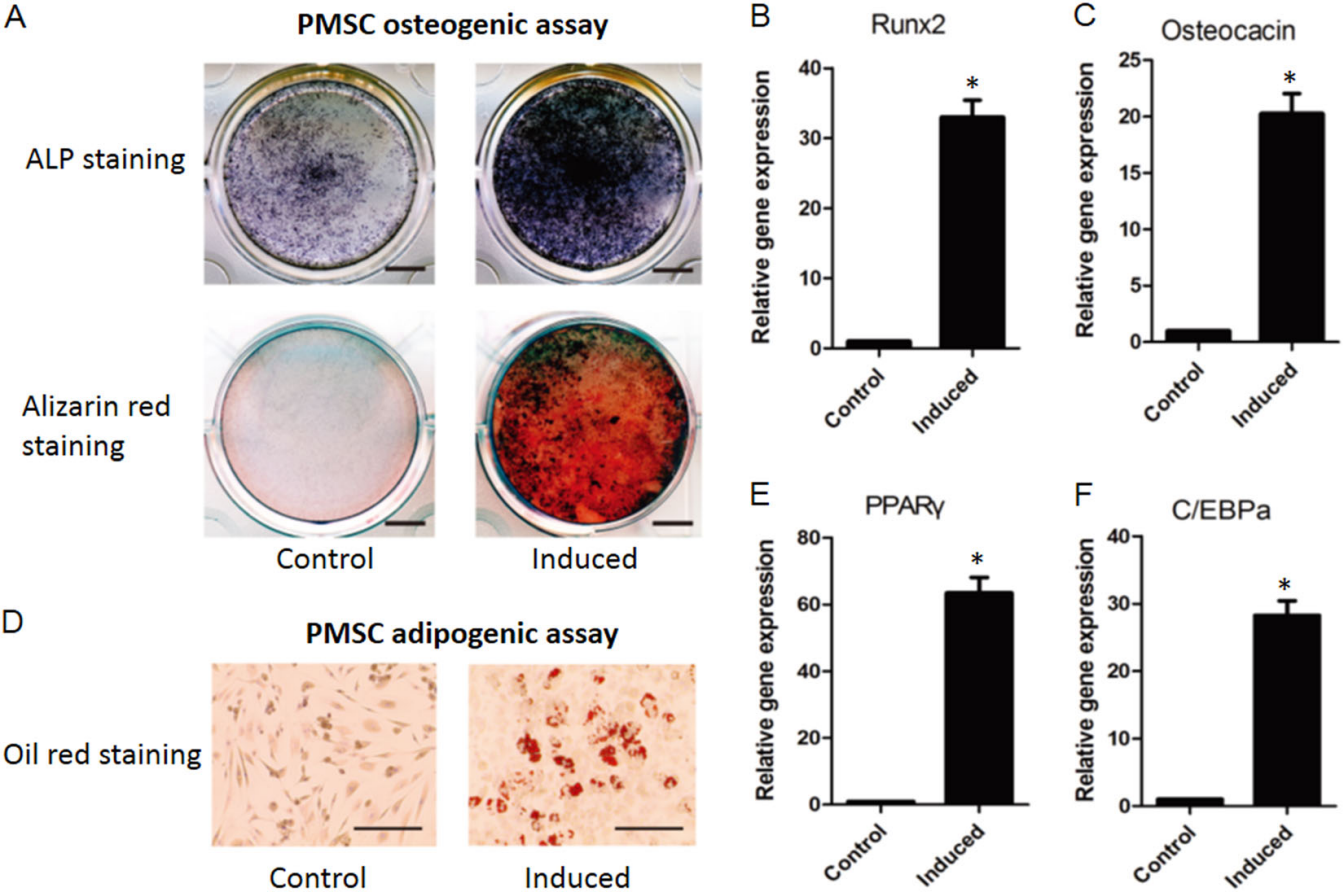
Analysis of PMSC osteogenic and adipogenic differentiation. **a** Osteogenic differentiation was examined by alkaline phosphatase staining and alizarin red staining without (Control) induction or with induction (Induced). Bar indicates 100 µm. **b**, **c** Osteogenic differentiation markers Runx2 and osteocalcin were further examined by real-time PCR. **d** Adipogenic differentiation was studied by the detection of lipid vacuoles by oil red O staining with or without induction. Bar indicates 50 µm. **e**, **f** Adipogenic differentiation markers PPARr and C/EBPa were inspected by real-time PCR. The data are the means ± SD of three independent experiments and all the results were normalized to the internal control (*p < 0.05 compared with control PMSCs without induction)

The isolation of PMSCs was performed following a procedure based on the adherence of cells to the surface of culture dishes. After 4 passages for expansion, most PMSCs showed a fibroblast-like morphology. Flow cytometry data indicated that the PMSCs were positive for stromal cell surface markers CD29, CD73, CD90, and CD166 (Fig. 1a–d), and negative for endothelial marker CD34 (Fig. 1e). The final quantification of these positive cell populations was further shown in Fig. 1f. More importantly, the results from in vitro differentiation assays clearly showed that these PMSCs could be successfully induced into osteoblastic cells as indicated by the enhanced ALP and alizarin red staining (Fig. 2a) when cultured in osteogenic medium for 14 days. PCR data confirmed enhanced osteogenesis by showing increased expression of Runx2 (Fig. 2b) and osteocalcin (OC) (Fig. 2c), which are both markers of osteoblasts. For adipogenic differentiation, intracellular lipid accumulation was noted in cells cultured with adipogenic medium (Fig. 2d), indicating that PMSCs could be differentiated into adipocytes in cultures. As expected, expression of adipogenic marker genes PPARr (Fig. 2e) and C/EBPa (Fig. 2f) were markedly increased in culture with adipogenic medium.

### Enhanced chondrogenic differentiation in PMSC culture with JAG1 treatment

**Fig. 3:**
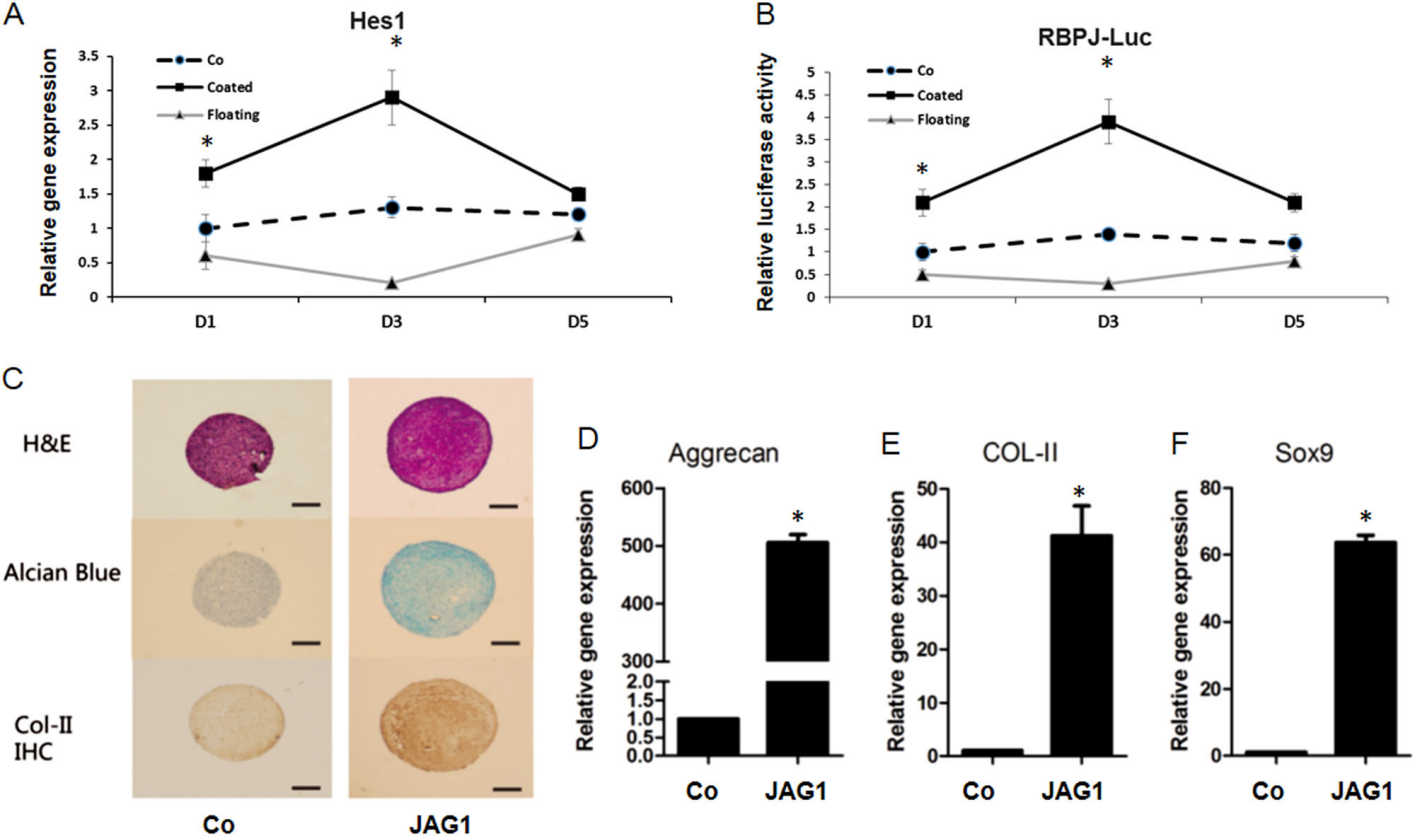
Inhibition of Notch signaling by unbound JAG1 enhances inducted PMSC chondrogenic differentiation. **a** Quantification of gene expression of the Notch target gene Hes1 indicates a significant increase (*p* < 0.05) in cells with surface-bound (coated) JAG1 (10 µg/ml) treatment, and a reduced expression was observed in cells treated with a single treatment of unbound (Floating) JAG1 (10 µg/ml) for up to 5 days. **b** Luciferase assays showed a significant decrease in Notch-responsive reporter activity in bound JAG1-treated MSCs and reduced activity in day 1 and day 3 with unbound JAG1 treatment. The data are the means ± SD of three independent experiments performed in duplicate, and the gene expression level in control cells (Co) was set at 1 (*p < 0.05 compared with control). **c** An increase in chondrogenesis was observed in unbound JAG1-treated PMSCs at day 14, with stronger staining of alcian blue and type II collagen (Col-II). Scale bars, 100 µm. **d**–**f** Quantification of gene expression of cartilage matrix aggrecan and Col-II, as well as transcription factor Sox9, using RNA from day 14 culture pellets. Data are the means ± SD of three independent experiments performed in triplicate, and all the results were normalized to internal control (*p < 0.05 compared with control PMSCs (Co)

To further investigate the effects of JAG1 on chondrogenic potential in PMSCs, a cell pellet culture assay was performed. Previous findings indicated that soluble extracellular forms of JAG1 DSL ligands can function to regulate Notch signal transduction either positively or negatively, depending on the context^17^. As reported, surface-bound JAG1 has been shown to activate Notch signaling in vitro, while, in contrast, floating ligands in medium inhibit Notch signaling by competing with membrane-bounded DSL ligands for Notch receptors^18^. To verify the different effects of surface-bound and unbound JAG1 on Notch signaling in PMSCs, we cultured cells in either JAG1-coated plates or plates containing soluble JAG1 in the medium for up to 5 days and then monitored the Notch activation by PCR and luciferase assay. Since our data showed that surface-bound JAG1 at 10 µg/ml can more effectively activate Notch signaling in MSCs than concentrations at 5 µg/ml and 15 µg/ml^12^, we decided to use 10 µg/ml JAG1 to treat cells in our subsequent experiments. Consistent with the previously finding, surface-bound JAG1 significantly induced expression of the Notch target Hes1 at day 1 in culture, and the expression peak was observed at day 3, followed by a decrease at day 5. In contrast, the addition of JAG1 in the medium resulted in a reduced expression of Hes1 at day 1 and a greater reduction at day 3 (Fig. 3a). Similar to the PCR data, the Notch signaling-responsive reporter (RBPJ-Luc) activity in PMSCs was also significantly enhanced by bound JAG1 and reduced by unbound JAG1 (Fig. 3b) for up to 5 days after a single treatment, confirming that unbound soluble JAG1 is capable of inhibiting Notch signaling in PMSCs.

To further explore the effect of unbound JAG1 on PMSC chondrogenic differentiation, we performed histological and immuno-staining on pellet cultures treated with chondrogenic medium containing either lgG or JAG1 for 14 days. Interestingly, treatment with soluble unbound JAG1 formed a larger pellet and induced stronger alcian blue staining than treatment with lgG peptides. Enhanced Col-II immune-staining further demonstrated that more cartilage matrix was synthesized in the JAG1-treated pellets than IgG-treated pellets (Fig. 3c). RT-PCR data showed significantly enhanced gene expression of chondrogenic markers, aggrecan (Fig. 3d) and Col-II (Fig. 3e) compared to the expression in control cells. Finally, a significantly increased expression of Sox9, a critical chondrogenic inducer, was observed in the JAG1-treated cell pellets compared to that in the IgG-treated cell pellets (Fig. 3f). Together, these data suggest that inhibition of Notch signaling by the unbound JAG1 ligand further promotes the PMSC chondrogenic differentiation induced by specific chondrogenic medium.

### JAG1-treated PMSCs induce cartilage repair in experimental OA

Having identified a clear induction of PMSC chondrogenesis by unbound JAG1, we next tested whether JAG1-treated PMSCs (JAG/PMSCs) could be utilized to enhance cartilage regeneration in a mouse OA model. To create an OA mouse model, MLI surgery was performed in 12-week-old mice. In this-modified experimental OA model, an OA-like phenotype in knee joints was rapidly induced as early as 4 weeks after surgery indicated by fibrillation, clefting and cartilage degradation since a portion of the meniscus was also removed^15^.

**Fig. 4:**
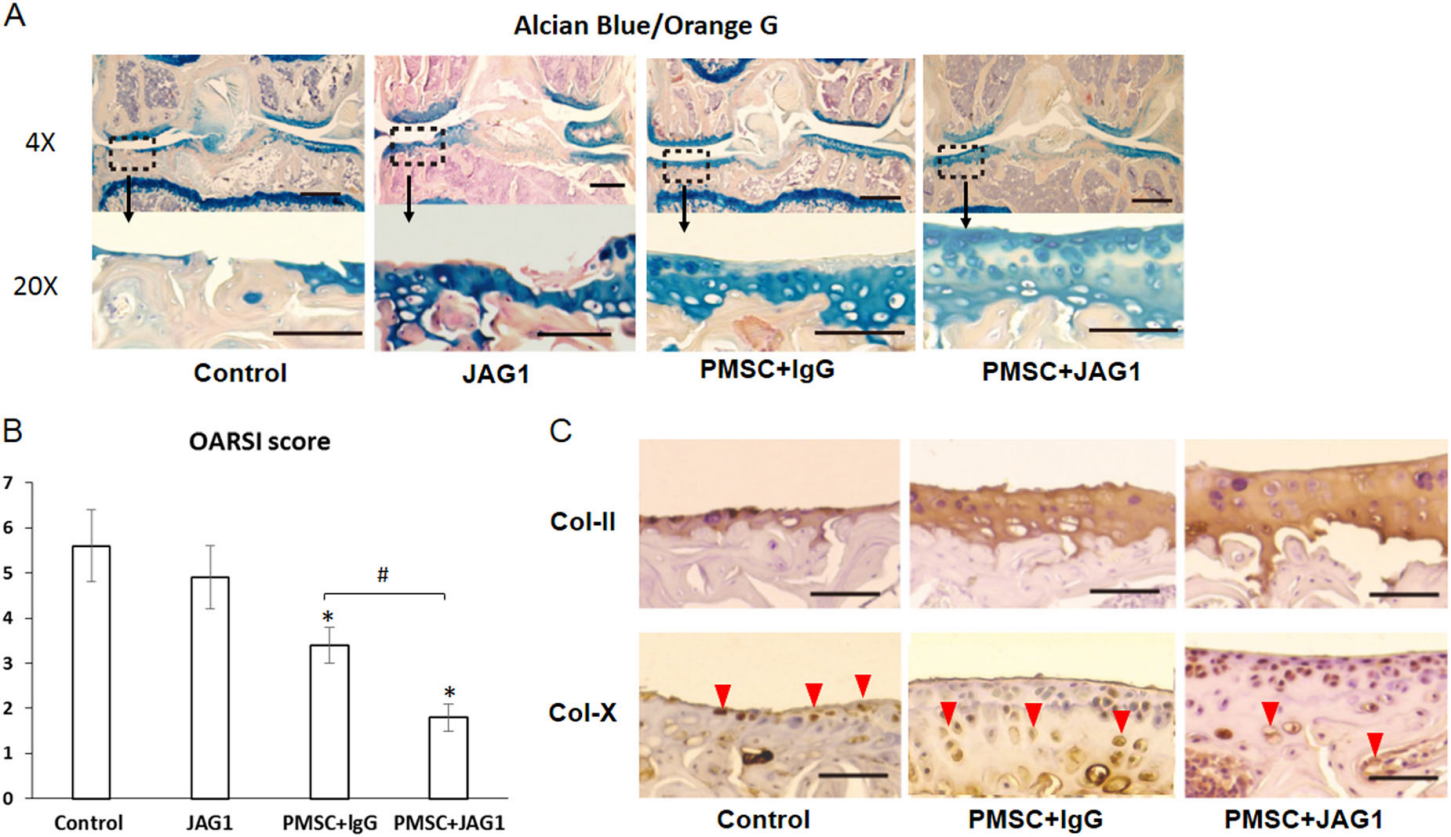
Mice develop more severe osteoarthritis without PMSC injection. At week 4 post-intra-articular injection of PBS, JAG1 (10 µg/ml) in PBS, PMSCs with lgG (10 µg/ml) or JAG1 (10 µg/ml), right knee joints were harvested for histological assessment. **a** Representative images of medial compartment of knee sections from MLI mice with injection of PBS (Control), JAG1, PMSC + lgG and PMSC + JAG1 stained with alcian blue/orange G. **b** Osteoarthritic changes in knee joints (*n* = 12) as quantified with Osteoarthritis Research Society International (OARSI) score. Data are the means ± SD (*p < 0.05 compared with control; #*P* < 0.05 between two groups). **c** Immunohistochemistry staining (IHC) of chondrogenic marker type II collagen (Col-ll) and type X collagen (Col-X) in tibia articular cartilage from 8-week MLI mice with or without PMSC injection. Increased Col-X expression was detected, and more Col-X-positive cells were located toward the articular surface in control and PMSC + lgG mice, not in PMSC + JAG1 mice 8-weeks after MLI surgery (Col-X positive cells: red arrowheads)

To study PMSC-mediated cartilage regeneration, intraknee joint injection of JAG1/PMSCs or lgG-treated PMSCs (lgG/PMSCs) was performed at 4 weeks after MLI surgery when the OA phenotype was established. To reduce the injection-stimulated joint swelling, we injected only a small amount of PMSCs (0.2 million) into OA joints starting at 4 weeks after MLI surgery. After four injections (once a week) in 4 weeks, the knee joints were then harvested for analysis. Consistent with previous findings, our histology staining (Fig. 4a) showed that severe OA-like defects were indeed developed in control mice at 8 weeks after MLI surgery in the PBS-injected group. Similar to the PBS control group, the JAG1 alone injection group also showed a severe loss of artificial cartilage with only a small alcian blue-positive area observed. In contrast, more alcian blue-positive cartilage was observed in the lgG/PMSC-injected groups than in the PBS or JAG1 alone-treated groups. More importantly, JAG1/PMSCs induced thicker cartilage formation than lgG/PMSCs, as shown by a wider alcian blue-positive top layer of cartilage. Consistent with histological observations, the evaluation using the OARSI scoring system (the higher the score, the greater the articular cartilage degeneration) revealed that although there was no significant difference between the PBS and JAG1 alone groups, a significant reduction in cartilage degeneration was observed in mice with JAG1/PMSC injection when compared to that in the lgG/PMSC groups (Fig. 4b). Since no significant difference was observed in OARSI scores between the PBS and JAG1 alone injection groups in MLI mice, in subsequent experiments, only PBS control, lgG/PMSC and JAG1/PMSC groups were used to identify possible mechanisms.

Our IHC data showed an increased expression of Col-II in the cartilage from the JAG1/PMSC group compared to that in the lgG/PMSC group (Fig. 4c), indicating that JAG1 induces more cartilage matrix formation when combined with PMSCs. Interestingly, a strong expression of type X collagen (a marker of chondrocyte hypertrophic transformation) was observed in the top layer of cartilage with injection of lgG/PMSCs. However, in mice with injection of JAG1/PMSCs, Col-X expression was only observed in the deep zone of articular cartilage (Fig. 4c), suggesting that while PMSC alone enhances cartilage formation, it fails to prevent chondrocyte terminal differentiation. In the control mice, since the superficial and mid zone of the cartilage was no longer present at 8 weeks after MLI surgery, Col-X expression at this time point was observed only in the thin surface layer, which is the exposed deep zone of the original articular cartilage.

### JAG1 treatment enhances PMSC survival and chondrogenic differentiation in vivo

**Fig. 5:**
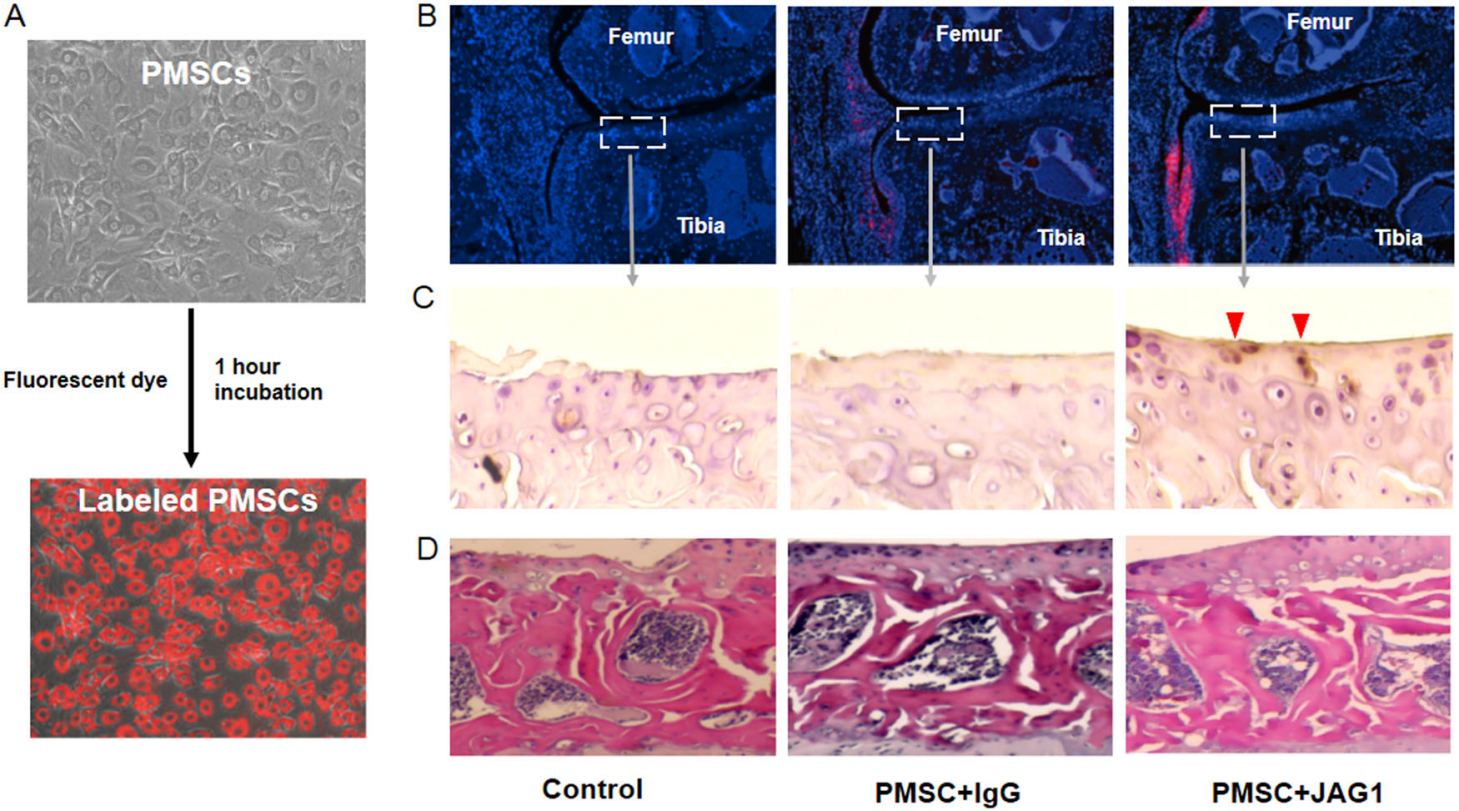
Tracing of PMSCs in the mouse knee joint. **a** Qtracker® reagent-labeled PMSCs showed strong red fluorescence after 1 h of incubation in the culture. **b** Mouse knee joints injected with 0.2 million red fluorescent-labeled JAG1-treated PMSCs showed more live PMSCs invading synovial tissue surrounding the artificial cartilage than joints injected with untreated PMSCs at 4 weeks after cell injection. **c** Immunohistochemistry staining (IHC) of human PMSCs using the anti-human nuclear antigen antibody in tibia articular cartilage from 8-week MLI mice (human nuclear antigen positive cells: red arrowheads). **d** H&E staining of subchondral bone in the mouse knee joint from control, PMSC and JAG1/PMSC groups at 4 weeks after cell injection

To track the in vivo engraftment and survival of PMSCs, cells were first labeled with red fluorescent dye that can be traced through several generations. After a one-hour incubation, cell labeling showed that 99% of PMSCs were successfully labeled with red dye without any noticeable cell death (Fig. 5a). More importantly, knee joint tissues from the mice with JAG1/PMSC injection had more red fluorescent signals remaining in the surgery joints 4 weeks after injection than the lgG/PMSC-injected surgery joints, suggesting that there were more live cells in the JAG1/PMSC-injected groups since the dead cells do not show red fluorescence (Fig. 5b). Interestingly, these labeled live PMSCs were only observed in the space between the tibia plateau and synovial membrane, not on the cartilage surface or inside of the artificial cartilage. Since fluorescent-labeled PMSCs also lose signal after extensive proliferation and cell differentiation, we further traced differentiated PMSCs using the anti-human nuclear antigen antibody. Our IHC data clearly showed that no positive cells were observed on the surface of cartilage in lgG/PMSC-injected mice. However, a number of positive cells were found on the top layer of the artificial cartilage in mice given injection of JAG1/PMSCs (Fig. 5c). Finally, our H&E staining of the tibia plateau showed no significant difference in the structure or density of subchondral bone among mice from the three different treatment groups (Fig. 5d), suggesting that neither PMSCs nor JAG1 had an effect on deeper-layer subchondral bone with short-term treatments.

### JAG1-treated PMSCs inhibit chondrocyte apoptosis and inflammation in mouse OA joint

**Fig. 6:**
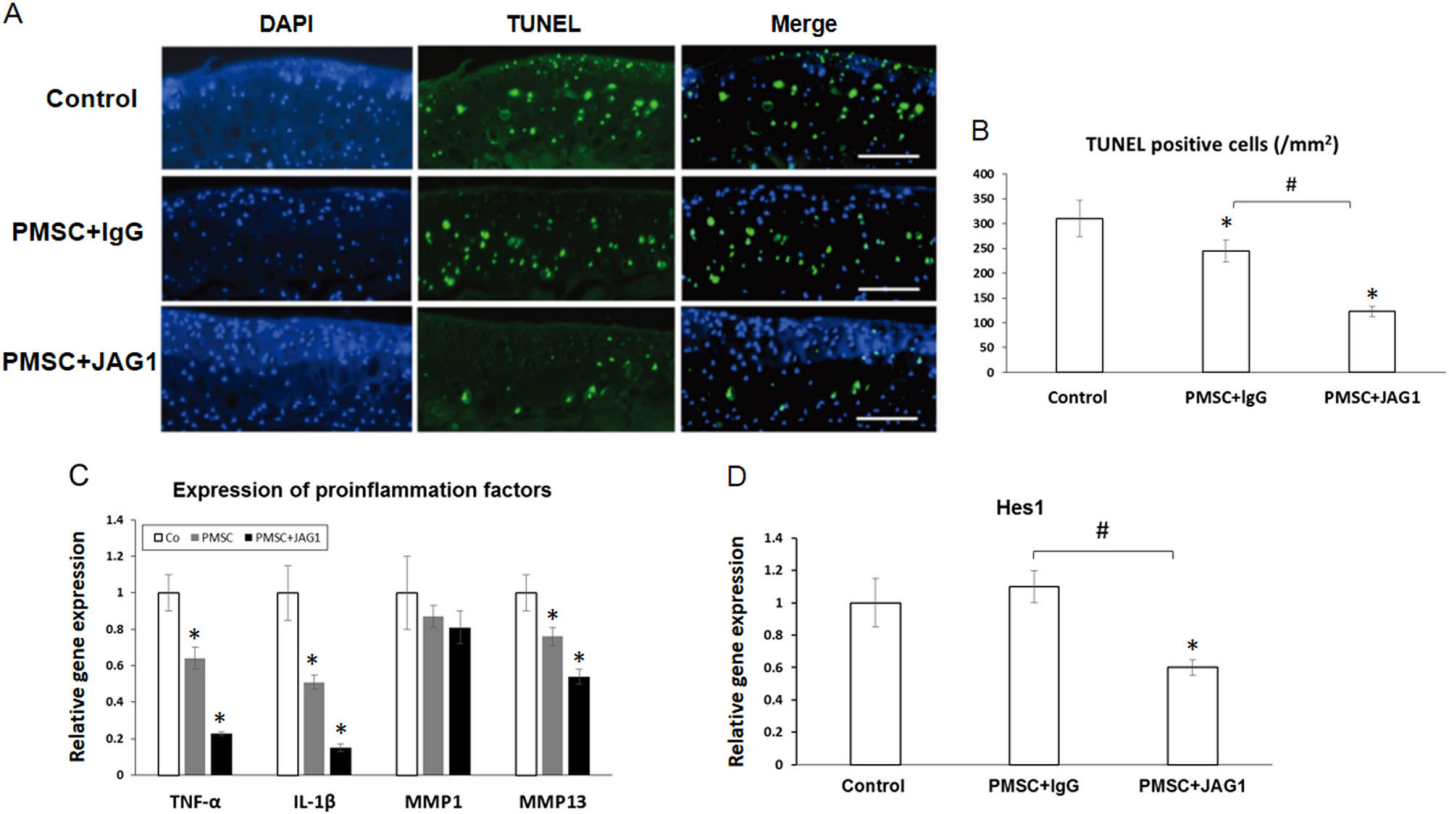
JAG1 treatment inhibits chondrocyte apoptosis and inflammation in the mouse OA joint. **a** Top panel: The representative images of TUNEL staining of the tibia plateau from a control OA mouse injected with 8 µl PBS. Middle panel: The stained tibia plateau from a control OA mouse injected with 8 µl PBS containing 0.2 million PMSCs and lgG (10 µg/ml). Low panel: The stained tibia plateau from a control OA mouse injected with 8 µl PBS containing 0.2 million PMSCs and JAG1 (10 µg/ml). **b** Quantification of TUNEL-positive cells in areas of the stained tibia plateau (*n* = 12). Data are the means ± SD (*p < 0.05 compared with control; #*P* < 0.05 between two groups). **c** Relative mRNA expression of proinflammation cytokines was analyzed by quantitative real-time PCR after synovial tissue obtained from OA mice was treated with PMSCs with or without JAG1 for 4 weeks. The data are the means ± SD (*p < 0.05 compared with control). **d** Relative mRNA expression of the Notch target gene Hes1 in synovial tissue was analyzed by quantitative real-time PCR. Data are the means ± SD (*p < 0.05 compared with control; #*P* < 0.05 between two groups)

Since the pathogenic mechanism of OA involves accelerated chondrocyte apoptosis, and the extent of chondrocyte apoptosis is correlated with severity of OA^19^, we next investigated the possible role of PMSCs in modulating apoptosis in OA articular cartilage. Our TUNEL staining data clearly showed that lgG/PMSC injection significantly reduced the chondrocyte apoptosis that was induced by MLI surgery in articular cartilage (Fig. 6a). More importantly, injection of JAG1/PMSCs led to an even greater reduction in chondrocyte apoptosis than injection of lgG/PMSCs (Fig. 6b). Since synovial tissue-produced proinflammatory cytokines, such as TNF-α, IL-1β, matrix metalloproteinase 1 (MMP1) and matrix metalloproteinase 13 (MMP13), have been shown to initiate local inflammatory responses and lead to the progression of cartilage cell apoptosis in the pathogenesis of OA^14^, we further measured the expression of these proinflammatory factors using RNA extracted from the synovial membrane surrounding the OA knee joint. Our PCR data showed that while no significant change was noticed in the expression of MMP1, a protein involved in the breakdown of extracellular matrix in normal physiological processes, inhibition of TNF-α, IL-1β, and MMP13 was observed with PMSC injection, and this inhibition was further potentiated by the addition of JAG1 (Fig. 6c). In addition, the expression of the Notch target gene Hes1 in synovial tissue was also significantly reduced by treatments with JAG1, suggesting decreased Notch activity in synovial tissues (Fig. 6d).

## Discussion

Osteoarthritis is a common joint disease, characterized by the degeneration of articular cartilage, that affects nearly 40 million people in the US alone^8^. Due to the lack of blood supply, joint cartilage has limited repair potential. Therefore, using an artificial joint to replace the damaged joint has been widely practiced in clinical settings to treat patients with severe cartilage loss^20^. Although joint replacement significantly improves the quality of life, limited activity and long-term joint loosening and revision make the new joint incomparable to the original joint. As such, disease-modifying treatments that effectively regenerate missing cartilage rather than replacing it are necessary for osteoarthritic patients.

To rapidly regenerate cartilage, one exciting strategy involves the use of multipotent MSCs. Since MSCs are known to home to diseased tissue and are preferentially attracted to diseased tissue rather than to intact tissue^4^, intra-articular injection of MSCs has been widely used to deliver cells to joints^21^. Although most of these clinical trials used autologous MSCs to eliminate immune rejection, several studies have attempted to investigate the potential application of allogeneic MSCs since these cells possess low immunogenicity^22^. In this study, MSCs isolated from human placentas were first characterized for multipotency. Our in vitro differentiation assays clearly showed that these cells could be differentiated into osteoblasts, chondrocytes, and adipocytes, which makes PMSCs an ideal cell source for tissue engineering and regenerative medicine. To further induce rapid chondrogenic differentiation of PMSCs, we utilized our recent finding that inhibiting Notch signaling promotes MSC differentiation for accelerated chondrogenesis^6^. Consistent with previous reports^11^, our data clearly showed that the activity of endogenous Notch signaling was successfully reduced by treatment with unbound JAG1 in PMSC pellet cultures. More importantly, PMSC chondrogenic differentiation was significantly enhanced by JAG1. Similar results were also observed by other research groups that showed that surface-bound JAG1-mediated Notch activation is able to suppress the default chondrogenic fate of vascular smooth muscle cell in cultures by repression of Sox9^23^. Additionally, adenoviral JAG1 transduction of human MSCs, which caused continuous expression of JAG1 and sustained Notch signaling, completely blocked chondrogenesis^24^, suggesting that inhibition of Notch signaling is critical for MSC chondrogenic differentiation. Therefore, we propose that once MSC chondrogenic differentiation is initiated, further Notch inhibition is required for enhanced chondrogenesis.

Although soluble JAG1 is able to induce rapid PMSC chondrogenesis ex vivo, whether it can be translated to a treatment for cartilage repair is not known. Since MLI surgery induces local inflammation and progressive cartilage cell death in the mouse knee joint, we further determined whether injection of JAG1-treated PMSCs would reduce or reverse the progression of OA in this mouse model. On the basis of the functional life (3–5 days) of JAG1 in culture, four intra-articular injections (once a week) were performed. This injection strategy not only induced several transient Notch inhibitions, which promoted the initiation of chondrogenic differentiation of injected PMSCs while also avoiding the possible side effects caused by constitutive Notch inhibition. Our results showed that cartilage loss was significantly ameliorated with the injection of JAG1-treated PMSCs, while injection of JAG1 alone did not exhibit any protective effects against OA progression, suggesting that PMSCs are required for the JAG1 effect on cartilage repair.

During OA progression, articular chondrocytes undergo hypertrophy-mediated cell death, and Col-X is a hallmark of this process^25^. In control mice with MLI surgery, an obvious increase in Col-X expression was observed in the thin top layer adjacent to the subchondral bone. Interestingly, JAG1-treated PMSCs showed a stronger inhibition of Col-X expression in both the superficial and middle zones of the cartilage than lgG-treated PMSCs, indicating that JAG1 treatment further potentiates the anti-hypertrophic effect of PMSCs. Furthermore, our in vivo cell tracking data indicated that most of the transplanted PMSCs were observed inside the inner layer of synovial tissues, not on the surface of the cartilage, suggesting that the distribution of PMSCs prefers soft synovial tissue rather than damaged, hard articular cartilage. Although PMSCs alone also enhanced cartilage repair, no human-derived cells were observed inside the cartilage, indicating that direct PMSC chondrogenic differentiation was not involved in this repair process. In contrast, we observed a small number of human cells embedded in the cartilage with the injection of JAG1-treated PMSCs, demonstrating that JAG1 can enhance both PMSC in vivo engrafting and subsequent chondrogenic differentiation.

Since JAG1-treated PMSCs survive longer in vivo than lgG-treated PMSCs, paracrine mechanisms mediated by PMSCs may also play an essential role in this reparative process. Previous studies have demonstrated that cytokines and growth factors from MSCs benefit tissue regeneration by anti-inflammatory and anti-apoptotic effects^26,27^. Supporting this notion, the cell apoptosis of articular chondrocytes in MLI-induced OA mice was significantly reduced by the injection of PMSCs, strongly suggesting that secreted factors from PMSCs may enhance cell survival of endogenous cartilage cells after injury. More importantly, JAG1 treatment further enhanced this anti-apoptotic effect of PMSCs on cartilage, confirming that paracrine mechanisms are involved in the anti-osteoarthritis effect of PMSCs, and this effect could further be enhanced by the inhibition of Notch signaling via JAG1 treatment. Since more live cells (red fluorescent-labeled) and less proinflammatory factors were observed in the JAG1-treated PMSC groups, we speculate that a positive feedback loop was formed, in which JAG1 treatment enhanced PMSC survival, producing more paracrine effectors to inhibit MLI surgery-induced inflammation, thereby reducing local inflammation and further promoting PMSC survival.

In the present study, we showed that PMSC local injection inhibits injury-induced cartilage destruction in mouse knee joints by inhibiting local inflammation and chondrocyte apoptosis. Treatment of PMSCs with the soluble Notch peptide, JAG1, further promotes its therapeutic effects via enhanced PMSC survival and chondrogenic differentiation, as well as reduced local inflammation.

## Disclaimer

The funders had no role in study design, data collection and analysis, decision to publish, or preparation of the manuscript.
